# Genome Sequences of an H5N1 Highly Pathogenic Avian Influenza Virus Isolated from Vaccinated Layers in China in 2012

**DOI:** 10.1128/genomeA.00936-13

**Published:** 2013-11-21

**Authors:** Hualei Liu, Xiaoliang Wang, Jingjing Wang, Yunling Zhao, Dongxia Zheng, Jiming Chen, Baoxu Huang, Zhiliang Wang

**Affiliations:** China Animal Health and Epidemiology Center, Qingdao, Chinaa; Ningxia Animal Disease Prevention and Control Center, Yinchuan, Chinab

## Abstract

An H5N1 virus was isolated from vaccinated layers during an outbreak of highly pathogenic avian influenza (HPAI) in Ningxia, China, in 2012. Phylogenetic analysis revealed that the virus is a novel variant in clade 7.2, and the outbreak likely resulted from mutations in the viral hemagglutinin (HA) gene.

## GENOME ANNOUNCEMENT

Highly pathogenic avian influenza (HPAI) viruses of the H5N1 subtype have caused serious outbreaks in poultry in multiple countries, including China, in recent years, and they pose a significant threat to human health (1, 2). Outbreaks of HPAI H5N1 viruses have declined dramatically in China since the implementation of the compulsory mass vaccination policy in 2004 (2, 3). However, the viruses have continued to circulate in China and have evolved into three main clades, namely clades 2.3.2, 2.3.4, and 7.2 (3, 4).

The first outbreak of an HPAI H5N1 virus in Ningxia, China, occurred in 2006, caused by a variant of H5N1 viruses in clade 7.2 (2, 3). Later, an antigen-matching vaccine designated the Re-4 vaccine was developed, and the vaccine has been used widely to prevent HPAI H5N1 viruses in the region since 2006 (2, 3). In April 2012, another serious outbreak of an HPAI H5N1 virus was confirmed in vaccinated layers in Ningxia, and a total of 95,000 birds were slaughtered to control the outbreak. One HPAI H5N1 virus strain, A/chicken/Ningxia/224/2012 (H5N1), was isolated from the infected layers. We amplified each segment of the viral genome using the method reported previously (5) and then sequenced the whole genome of the virus through the routine Sanger sequencing technology.

The viral hemagglutinin (HA) gene contains multiple basic amino acids adjacent to the cleavage site (RRRKR↓GLF), which represents the high pathogenicity of the virus in poultry (6). The receptor binding site in the viral HA gene possesses the residues Q226 and G228 (H3 numbering), indicating the preferential binding of the virus to avian-like receptors (7). The matrix (M) gene sequence of the isolate contains the S31N mutation, which might confer amantadine resistance (8).

Phylogenetic analysis indicated that the virus belongs to clade 7.2. All the viral genomic segments share high homology (>96%) in their nucleotide sequences with another virus strain in clade 7.2 isolated in 2009, A/chicken/Hebei/A-8/2009 (H5N1). The nucleotide sequence of the viral HA gene only shares 94.1% homology with that of A/chicken/Shanxi/2/2006 (H5N1), which is the prototype strain of clade 7.2 and one parent of the corresponding Re-4 vaccine strain.

Between the novel isolate A/chicken/Ningxia/224/2012 (H5N1) and the vaccine strain Re-4, some critical amino acid substitutions in the viral HA1 domain occurred at antigenic sites, such as K53E, K115E, S121H, E126N, A127T, G139E, K140N, T167A, D183N, K189M, and T195N (H5 numbering), which can change the antigenicity of the viruses (9, 10).

### Nucleotide sequence accession numbers.

The genome sequences of A/chicken/Ningxia/222/2012(H5N1) have been deposited in GenBank under accession no. KF638576 to KF638583.
